# Single-Cell RNA Sequencing Reveals the Diversity of the Immunological Landscape following Central Nervous System Infection by a Murine Coronavirus

**DOI:** 10.1128/JVI.01295-20

**Published:** 2020-11-23

**Authors:** Amber R. Syage, H. Atakan Ekiz, Dominic D. Skinner, Colleen Stone, Ryan M. O’Connell, Thomas E. Lane

**Affiliations:** a Division of Microbiology & Immunology, Department of Pathology, School of Medicine, University of Utah, Salt Lake City, Utah, USA; b Huntsman Cancer Institute, University of Utah, Salt Lake City, Utah, USA; Loyola University Chicago

**Keywords:** host defense, immunology, central nervous system, coronavirus, central nervous system infections, neuroimmunology, single-cell RNA sequencing

## Abstract

Intracranial (i.c.) infection of susceptible C57BL/6 mice with the neurotropic JHM strain of mouse hepatitis virus (JHMV) (a member of the *Coronaviridae* family) results in acute encephalomyelitis and viral persistence associated with an immune-mediated demyelinating disease. The present study was undertaken to better understand the molecular pathways evoked during innate and adaptive immune responses as well as the chronic demyelinating stage of disease in response to JHMV infection of the central nervous system (CNS). Using single-cell RNA sequencing analysis (scRNAseq) on flow-sorted CD45-positive (CD45^+^) cells enriched from brains and spinal cords of experimental mice, we demonstrate the heterogeneity of the immune response as determined by the presence of unique molecular signatures and pathways involved in effective antiviral host defense. Furthermore, we identify potential genes involved in contributing to demyelination as well as remyelination being expressed by both microglia and macrophages. Collectively, these findings emphasize the diversity of the immune responses and molecular networks at defined stages following viral infection of the CNS.

**IMPORTANCE** Understanding the immunological mechanisms contributing to both host defense and disease following viral infection of the CNS is of critical importance given the increasing number of viruses that are capable of infecting and replicating within the nervous system. With this in mind, the present study was undertaken to evaluate the molecular signatures of immune cells within the CNS at defined times following infection with a neuroadapted murine coronavirus using scRNAseq. This approach has revealed that the immunological landscape is diverse, with numerous immune cell subsets expressing distinct mRNA expression profiles that are, in part, dictated by the stage of infection. In addition, these findings reveal new insight into cellular pathways contributing to control of viral replication as well as to neurologic disease.

## Abstract (precis)

Understanding the immunological mechanisms contributing to both host defense and disease following viral infection of the CNS is of critical importance given the increasing number of viruses that are capable of infecting and replicating within the nervous system. With this in mind, the present study was undertaken to evaluate the molecular signatures of immune cells within the CNS at defined times following infection with a neuroadapted murine coronavirus using scRNAseq. This approach has revealed that the immunological landscape is diverse, with numerous immune cell subsets expressing distinct mRNA expression profiles that are, in part, dictated by the stage of infection. In addition, these findings reveal new insight into cellular pathways contributing to control of viral replication as well as to neurologic disease.

## INTRODUCTION

Intracranial infection of susceptible mice with the neurotropic JHM strain of mouse hepatitis virus (JHMV) provides an excellent model in which to interrogate molecular and cellular mechanisms of host defense and disease in response to viral infection of the central nervous system (CNS) (1, 2). JHMV is a member of the betacoronavirus subfamily and is related to severe acute respiratory syndrome coronavirus (SARS-CoV) and Middle East respiratory syndrome-CoV (MERS-CoV) and to the recently described SARS-CoV-2, which rapidly spread to pandemic status in early 2020 (3). JHMV infection results in acute encephalomyelitis characterized by viral replication in astrocytes, microglia (MG), and oligodendrocytes with relative sparing of neurons (4). Following JHMV infection of the CNS, there is orchestrated expression of cytokines and chemokines that results in targeted recruitment of cells of both the innate and adaptive immune responses (5–20). Virus-specific CD4-positive (CD4^+^) and CD8^+^ T cells infiltrate the CNS and control JHMV replication through gamma interferon (IFN-γ) secretion and perforin-mediated cytolytic activity (21, 22). Although a robust cell-mediated immune response occurs during acute disease, sterilizing immunity is not achieved, resulting in viral persistence in white matter tracts (23). Ultimately, virus-specific antibody is critical for additional control of viral replication and suppression of viral recrudescence (24). Histological features associated with viral persistence include the development of an immune-mediated demyelinating disease that is similar to the human demyelinating disease multiple sclerosis (MS), with infiltrating T cells and macrophages amplifying white matter damage (8, 17, 25).

Although the previous studies on bulk cell populations revealed important information on the mechanisms of disease and host defense in JHMV-infected mice, characterization of molecular alterations at the single-cell level is lacking. In this study, we employed single-cell RNA sequencing (scRNAseq) on enriched CD45-positive (CD45^+^) cells isolated from brains and spinal cords at defined times following JHMV infection to delineate mechanisms associated with effective host responses as well as those that contribute to both demyelination and remyelination within the CNS.

## RESULTS

### Intracranial infection of mice with JHMV.

**FIG 1:**
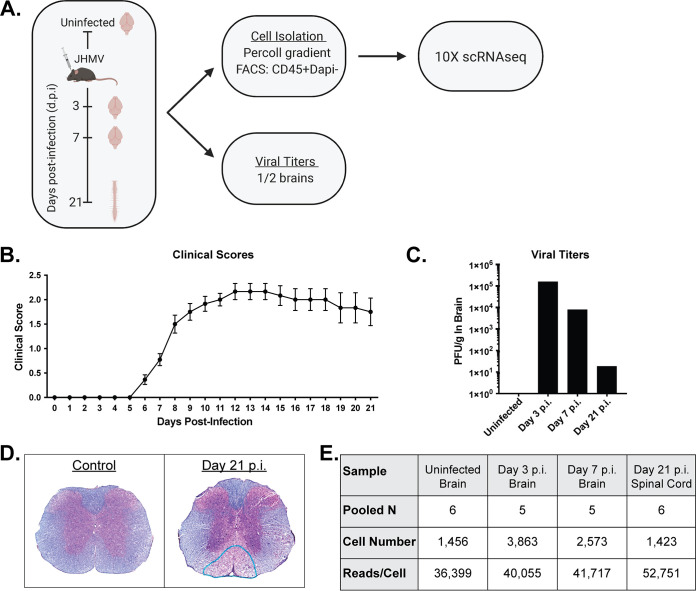
JHMV infection of the CNS. (A) Schematic outline of experimental design, indicating times postinfection (p.i.) for sacrifice and for tissue isolation and experimental procedures performed. (B) Clinical disease in C57BL/6 mice (*n *= 22) following intracranial (i.c.) inoculation with 1,500 PFU of JHMV. (C) Viral titers within the brains from uninfected mice and infected animals at days 3, 7, and 21 p.i. (*n *= 5 to 6 per group) (D) Representative spinal cords stained with hematoxylin and eosin (H&E) and Luxol fast blue (LFB) from uninfected control mice and infected mice at day 21 p.i., revealing demyelination within the ventral funiculus and lateral white matter columns. (E) Data table summarizing the number of CD45^+^ cells isolated from either brain (control and days 3 and 7 p.i.) or spinal cord (day 21 p.i.) tissue used in downstream analysis as well as the number of reads/cell following scRNAseq analysis (5 to 6 mice pooled per group).

Adult (5-to-6-week-old) C57BL/6 mice were intracranially (i.c.) infected with 1,500 PFU of JHMV. At early time points following CNS infection, JHMV initiates replication within the brain and triggers a strong host immune response before ultimately spreading into the spinal cord, causing immune-mediated demyelination. Therefore, we chose to examine brains at days 3 and 7 postinfection (p.i.) to investigate the immune landscape during innate and adaptive responses, respectively, and to examine spinal cords at day 21 p.i. to evaluate immune cell responses during chronic demyelination. CD45-positive (CD45^+^) cells were subjected to flow sorting (flow sorted) from brains of JHMV-infected mice at days 3 and 7 p.i. and spinal cords at day 21 p.i. (Fig. 1A). For controls, we flow sorted CD45^+^ cells from brains of uninfected C57BL/6 mice. Assessment of disease phenotype extending to day 21 p.i. showed that all experimental mice developed disease consistent with JHMV-induced neurologic disease (Fig. 1B). Furthermore, plaque assays conducted using the brains of infected mice showed viral titers peaking at day 3 p.i. and subsequently declining at days 7 and 21 p.i. (Fig. 1C). Viral titers within the spinal cords at day 21 p.i. were below the level of detection (∼100 PFU/g tissue, data not shown), which is consistent with previous studies (23, 26). By day 21 p.i., there was extensive spinal cord demyelination compared to spinal cords from noninfected control animals (Fig. 1D). As shown in Fig. 1E, single-cell RNA sequencing (scRNAseq) analysis was conducted on a total of 1,456 CD45^+^ cells from the brains of uninfected mice (*n* = 6, pooled), on 3,863 CD45^+^ cells at day 3 p.i. (*n* = 5, pooled), and on 2,573 CD45^+^ cells at day 7 p.i. (*n* = 5, pooled) from the brains of JHMV-infected mice and on 1,423 CD45^+^ cells from spinal cords at day 21 p.i. (*n* = 6, pooled) with JHMV.

### scRNAseq and immune cell diversity within the CNS of JHMV-infected mice.

**FIG 2:**
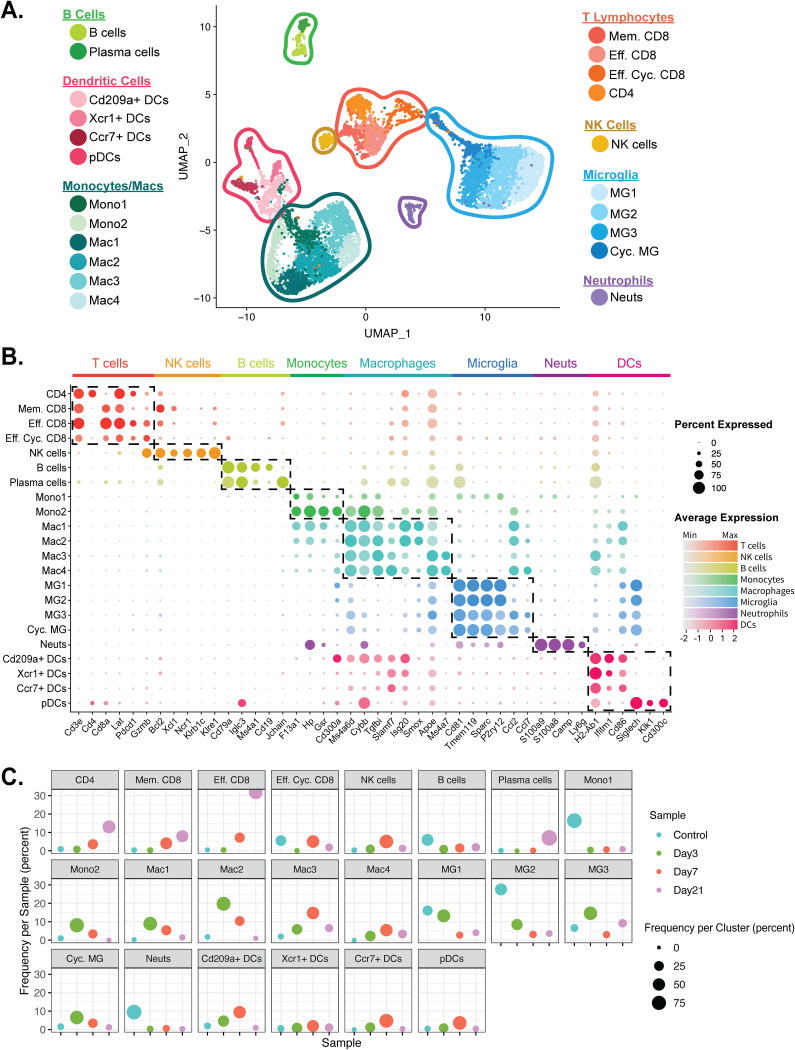
scRNAseq analysis of CD45^+^ cells within the CNS at defined times p.i. (A) UMAP plot showing aggregate data representing the immune landscape in brains and spinal cords of uninfected (Control) and infected mice at 3, 7, and 21 days p.i. with JHMV, revealing 22 distinct cell clusters (5 to 6 mice pooled per group). Neuts, neutrophils. (B) Dot plot presenting expression of selected genes within the 22 cell clusters. Dot size is representative of the frequency of cells within a cluster expressing the gene of interest, while the degree of color intensity is indicative of the level of expression of the gene. The dashed boxes highlight commonly and uniquely expressed genes of clusters within the overarching cell types. (C) Dot plot showing percent frequency of cells for each cluster. The *y* axis shows the overall frequency of cells from a cluster per sample, while dot size represents the frequency of cells from control (teal), day 3 p.i. (green), day 7 p.i. (red), and day 21 p.i. (purple) within a cell cluster.

Downstream analyses of processed data were performed on a combined total of 9,315 cells from brains and spinal cords of experimental (both uninfected and infected) mice via principal-component analysis (PCA) and uniform manifold approximation and projection (UMAP) dimensional reduction (27, 28). Unsupervised clustering revealed 7 different major populations with 22 different minor clusters (Fig. 2A). We identified two different monocyte clusters (Mono1 and Mono2); three unique CD8^+^ T cell clusters (effector [Eff.] CD8, memory [Mem.] CD8, and effector cycling [Eff. Cyc.] CD8); four dendritic cell (DC) populations (Cd209a^+^ [*Cd209a*], Ccr7^+^ [*Ccr7*], Xcr1^+^ [*Xcr1*], and plasmacytoid dendritic cells [pDCs]); four microglia clusters (MG1, MG2, MG3, and cycling MG); four macrophage populations (Mac1, Mac2, Mac3, and Mac4); two B cell clusters which included plasma cells; and single CD4 T cell, NK cell, and neutrophil populations (Fig. 2A). In order to verify the algorithm-assisted identification of cell clusters, we examined expression of known cellular markers in our data set; expression of these markers corresponded with the respective identities of the distinct cell clusters (Fig. 2B) (29, 30). Plotting data from different time points side by side, dynamic changes in the proportions of these cell populations following JHMV infection were found to begin to emerge (Fig. 2C). For example, we detected a drop in the frequency of neutrophils at day 3 p.i. compared to uninfected mice and these levels remained reduced through the course of infection. NK cells peaked at day 7 p.i., and cells from this time p.i. predominated this cluster. With regard to cells involved in antigen presentation, we observed increases in the frequencies of all DC subsets by day 3 p.i., with levels peaking within the brain at day 7 p.i. A similar trend was found in macrophage subpopulations Mac3 and Mac4. In contrast, frequencies of macrophage subsets Mac1 and Mac2 and monocytes peaked at day 3 p.i. and subsequently declined at days 7 and 21 p.i. Microglia subsets MG1 and MG2 were detectable in uninfected brains (control) and at day 3 p.i. but then were reduced in frequency at days 7 and 21 p.i. We also saw an overall trend in increased frequencies in T cell subsets, with the majority of these subsets peaking within the spinal cords of infected mice at day 21 p.i. Frequencies of infiltrating B cells within the CNS remained relatively constant, and yet the frequencies of plasma cells peaked at day 21 p.i.

### Genetic signatures of immune cell subsets within the CNS of JHMV-infected mice.

**FIG 3:**
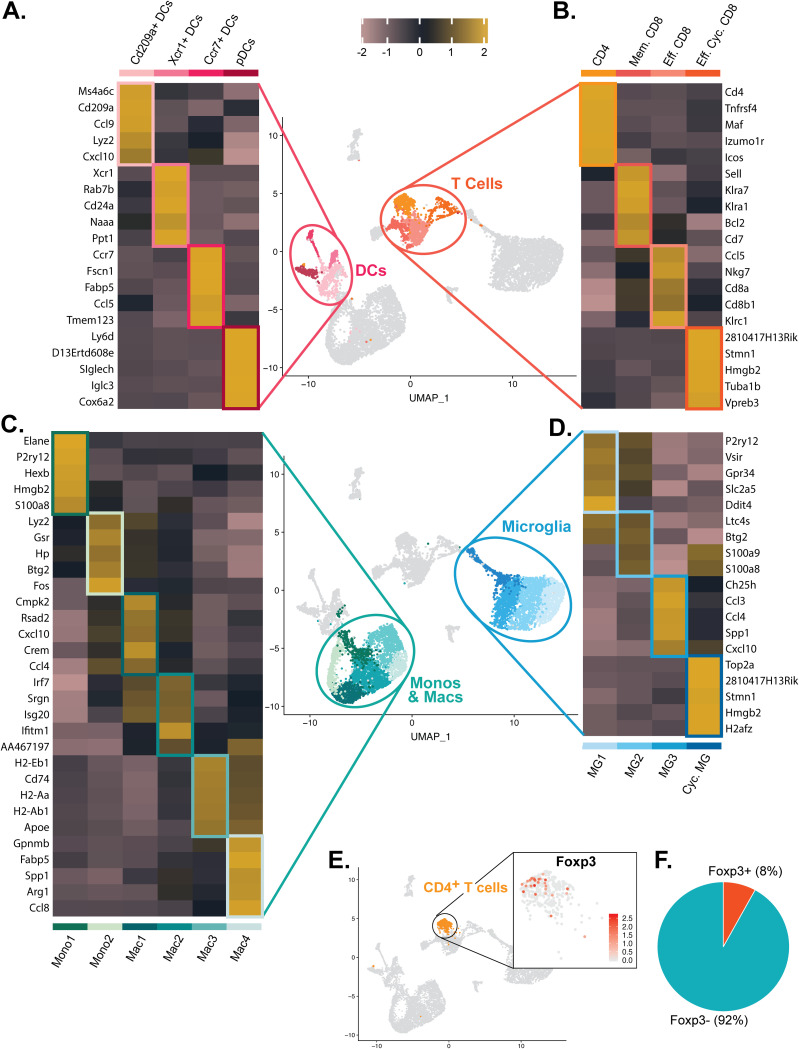
Genetic signatures of immune cell subsets within the CNS of JHMV-infected mice. Heat maps show the top 5 transcripts differentially expressed between heterogeneous subpopulations for subsets of (A) dendritic cells, (B) T cells, (C) monocytes/macrophages, and (D) microglia. Each heat map was generated with subset data from (A) dendritic cells, (B) T cells, (C) monocytes/macrophages, and (D) microglia populations and isolated from all other clusters outside what is shown in each individual map. For panels A to D, data were aggregated from uninfected (Control) and infected mice at days 3, 7, and 21 p.i. Columns represent the different clusters, and rows represent expression of transcripts. (E) UMAP plot showing scaled expression of transcripts encoding FOXP3 (*Foxp3*) in CD4^+^ T cells. (F) Pie chart showing the percentage of *Foxp3*^+^ cells (red) versus *Foxp3*-negative (*Foxp3*-) cells (blue) within the CD4^+^ T cell cluster.

We were able to identify distinct subtypes of immune cells based upon unique genetic signatures associated with both defense and disease within the CNS of JHMV-infected mice. We identified four DC effector subtypes, including Cd209^+^ DCs, Xcr1^+^ DCs, Ccr7^+^ DCs, and plasmacytoid DCs (pDCs), that express a network of genes associated with effective antiviral responses (Fig. 3A). We identified one population of CD4^+^ T cells expressing transcripts encoding activation markers, including *Tnfrsf4* and *Icos* (Fig. 3B). We identified three different CD8^+^ T cell types (memory [Mem.] CD8, effector [Eff.] CD8, and effector cycling [Eff. Cyc.] CD8 cells), each a unique expression profile (Fig. 3B). Similarly, we identified two monocyte clusters, four macrophage clusters (Fig. 3C) and four microglia subsets (Fig. 3D) with unique genetic profiles. With regard to the single cluster of CD4^+^ T cells compared to the three clusters of CD8^+^ T cells, we were surprised that we did not see regulatory CD4^+^ T cells as previous studies had demonstrated the presence of these cells within the brains of JHMV-infected mice at day 7 p.i. (31). Subsequent analysis revealed that ∼8% of cells within the CD4^+^ T cell cluster expressed FoxP3 transcripts (Fig. 3E and F).

**FIG 4:**
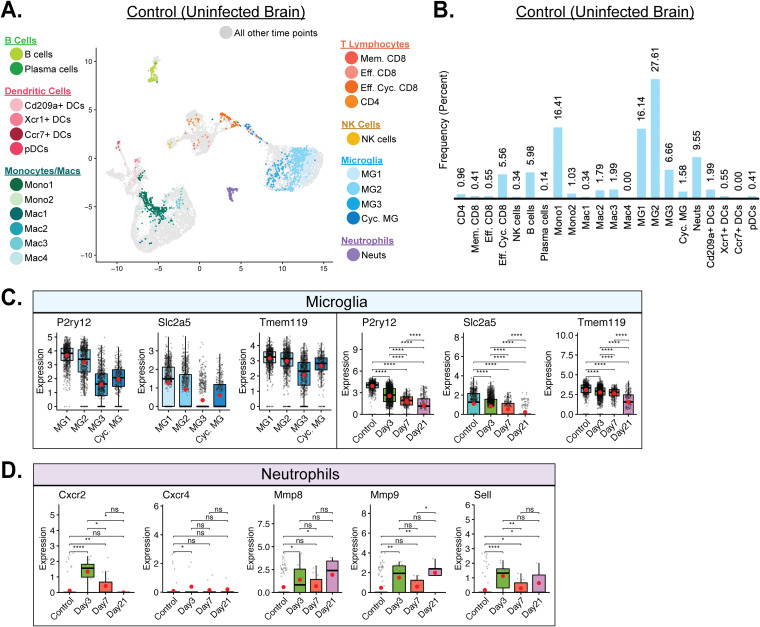
Immune landscape in the uninfected CNS. (A) UMAP plot of cells within clusters exclusively from uninfected control brains. (B) Bar graph showing frequency of cell clusters within the brains of uninfected mice. Microglia represent the dominant cell types enriched within uninfected brains, followed by the monocyte subpopulation Mono1 and neutrophils. (C) Expression of *P2ry12*, *Slc2a5*, and *Tmem119* transcripts by microglia (MG). Temporal analysis results represent aggregate data from subsets. (D) Temporal analysis of expression of transcripts encoding CXCR2/4 (*Cxcr2*/*Cxcr4*), MMP-8/9 (*Mmp8*, *Mmp9*), and l-selectin (CD62L, *Sell*), which are indicative of neutrophil activation. Transcript levels were elevated in defined times postinfection with JHMV. For the data presented in panels C and D, normalized expression values were used, and random noise was added to show the distribution of data points. The box plots show interquartile range and the median value (bold horizontal bar). Average expression value per sample is indicated by the red dots. Wilcoxon’s test was used for statistical analysis. ns, not significant; *, *P* ≤ 0.05; **, *P* ≤ 0.01; ****, *P* ≤ 0.0001.

Monocyte subtypes expressed *F13a1*, *Hp*, *Gsr*, and *Cd300a*, and while Mono2 appeared to overlap Mac1, Mono1 seemed to be representative of a steady-state immune surveilling population of monocytes, having elevated expression of *S100a8* (Fig. 3C) (32). The four different macrophage populations expressed a common macrophage marker, *Ms4a6d*, and displayed a transitional gradient from early to later defense against viral infection, with Mac1 having the highest expression of *Cybb* and lowest expression of *Tgfb1* and *Slamf7* and Mac4 showing the highest expression of *Slamf7* and *Tgfbi* and the lowest expression of *Cybb* (Fig. 2B). Mac1 and Mac2 both showed elevated expression of *Smox* and *Isg20* (Fig. 2B), and compared with subpopulations consisting only of monocytes and macrophages, Mac1 uniquely expressed *Cmpk2*, *Rsad2*, *Cxcl10*, *Crem*, and *Ccl4* whereas Mac2 was characterized by increased levels of IFN-related transcripts, including *Irf7*, *Isg20*, and *Ifitm1* (Fig. 3C). Interestingly, Mac3 and Mac4 presented a more phagocytic effecter phenotype, with both expressing *Apoe* and *Ms4a7* (Fig. 3C). Compared with the other subpopulations of monocytes and macrophages, Mac3 additionally expressed transcripts encoding major histocompatibility complex (MHC) class II molecules and F4/80 (*Adgre1*), while Mac4 uniquely expressed *Gpnmb*, *Fabp5*, *Spp1*, *Arg1*, and *Ccl8* (Fig. 3C). As for the microglia subpopulations, MG1 and MG2 appeared to be more “homeostatic,” showing higher levels of expression of *Tmem119*, *P2ry12*, and *Slc2a5* (Fig. 4C) (33). In contrast, MG3 and cycling (Cyc.) MG appeared to be more extensively disease associated, with higher levels of *Ccl2* and *Ccl7* (Fig. 2B) (13, 34). There was only one minor NK cell cluster, represented by expression of transcripts *Ncr1*, *Klrb1c*, *Klre1*, *Xcl1*, and *Bcl2* (Fig. 2B). B cells were subdivided into one minor cluster and a cluster of plasma cells. B cells were characterized by expression of *Cd79a*, *Iglc3*, *Ms4a1*, and *Cd19*, whereas plasma cells displayed high expression levels of *Cd79a* as well as *Jchain* and immunoglobulin transcripts, indicative of antibody secretion (35, 36) (Fig. 2B).

### Selected immune cells are present in the uninfected brain.

We next assessed the immunological landscape of the uninfected brain through the use of scRNAseq on flow-sorted CD45^+^ cells. This approach revealed that microglia subtypes MG2 and MG1, which made up 27.61% and 16.14% of the total CD45^+^ cells sequenced from the control sample, respectively, represented predominant immune cell subtypes within the brains of uninfected mice (Fig. 4B). MG1 and MG2 trended toward a more extensively homeostatic microglia subtype, with higher expression levels of *Tmem119*, *P2ry12*, and *Slc2a5* (Fig. 4C) (33). The Mono1 subpopulation of monocytes was also enriched within uninfected brains, contributing 16.41% of the total sample population (Fig. 4B). Neutrophils (9.55%), B cells (5.98%), and effector cycling CD8^+^ T cells (5.56%) were also among the other prevalent immune cell types present in the uninfected brains of mice (Fig. 4B). With regard to neutrophil populations within the brains of uninfected mice, in evaluating expression of transcripts encoding common activated neutrophil genes, including C-X-C motif chemokine receptor 2 and 4 (CXCR2/4; *Cxcr2* and *Cxcr4*), matrix metalloproteinase 8 (MMP8) and MMP9 (*Mmp8* and *Mmp9*), and l-selectin (CD62L; *Sell*), there was little to no expression of these activation transcripts in uninfected control mice and yet expression levels were increased at day 3 p.i. as well as at subsequent time points p.i. (14, 37–39) (Fig. 4D). These findings support the notion of low-level immune surveillance of the uninfected CNS (40–43) and emphasize the rapid mobilization of innate and adaptive immune responses following infection.

### Heterogeneous antiviral mechanisms are elicited in innate cell subsets within the CNS following JHMV infection.

**FIG 5:**
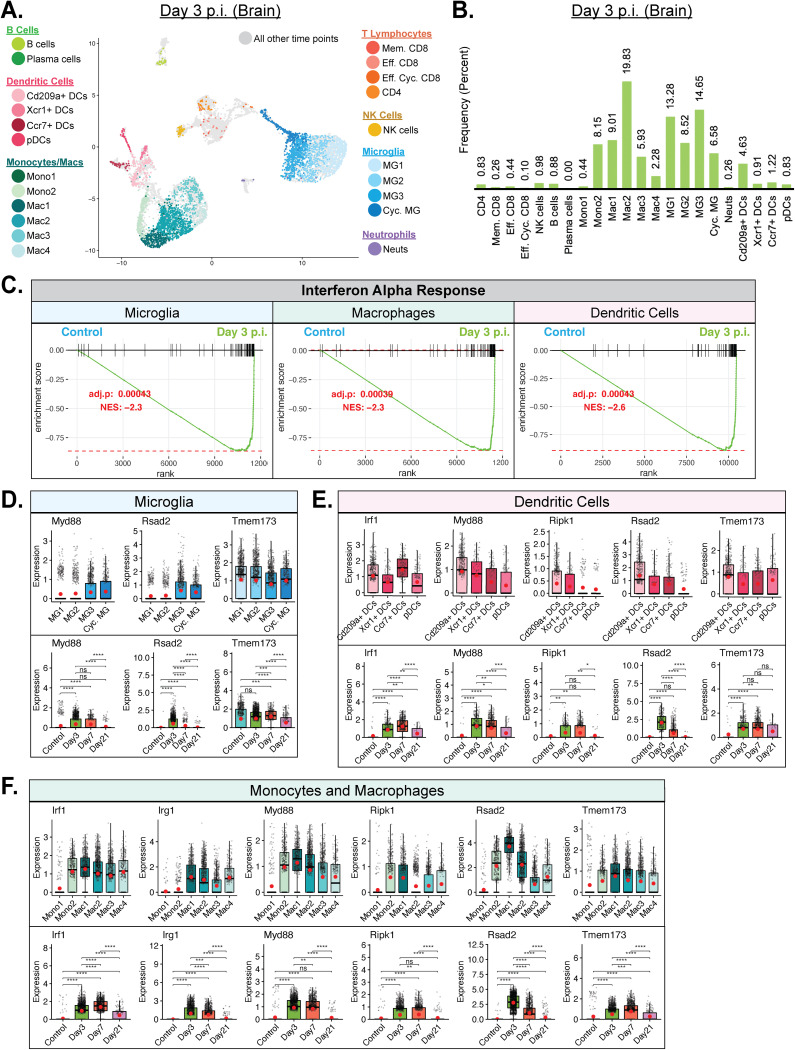
Innate immune response following JHMV infection of the CNS. (A) UMAP plot of cells within clusters exclusively from brains at day 3 p.i. with JHMV. (B) Bar graph showing frequency of cells from a cluster within the brains of mice 3 days postinfection (p.i.) with JHMV. Macrophages and microglia represented the main cell types enriched at day 3 p.i. (C) Gene set enrichment analysis (GSEA) for IFN-α responses in microglia, macrophages, and dendritic cells at day 3 p.i. from brains of JHMV-infected mice. The area under the curve represents enrichment of response genes. Normalized enrichment scores and *P* values are shown. (D) Data showing expression of *Myd88*, *Rsad2*, and *Tmem173* transcripts in microglia subpopulations as well as overall temporal expression at defined times p.i. with JHMV (E) Expression of *Irf1*, *Irg1*, *Myd88*, *Ripk1*, *Rsad2*, and *Tmem173* transcripts in subpopulations of monocytes (monos) and macrophages (macs) in response to JHMV infection. (F) Expression of *Irf1*, *Myd88*, *Ripk1*, *Rsad2*, and *Tmem173* in dendritic cell (DC) subpopulations following JHMV infection. Normalized expression values were used, and random noise was added. Box plots show interquartile range, median value (bold horizontal bar), and average expression value per sample (red dots). Wilcoxon’s test was used for statistical analysis. ns, not significant; *, *P* ≤ 0.05; **, *P* ≤ 0.01; ***, *P* ≤ 0.001; ****, *P* ≤ 0.0001.

Examining the immune responses at day 3 p.i. revealed increased subpopulations of monocytes (Mono2), macrophages (Mac1, Mac2, and Mac3), microglia (MG1, MG2, MG3, and Cyc. MG) and DCs (Cd209a^+^ DCs) (Fig. 5A and B). The most prominent of these populations included Mac2 (19.83%), MG3 (14.65%), and MG1 (13.28%) (Fig. 5B). Gene set enrichment analysis (GSEA) revealed increased interferon alpha (IFN-α) responses in microglia, macrophages and dendritic cells (Fig. 5C). Specifically, we found increased expression of transcripts encoding antiviral response factors, including *Myd88*, *Rsad2* (Viperin), and *Tmem173* (STING), which were enriched in distinct subpopulations of monocytes/macrophages (Fig. 5D), microglia (Fig. 5E), and DCs (Fig. 5F). For all three cell populations, there was an increase in expression of *Myd88* and *Rsad2* in the brains at days 3 and 7 p.i. compared to control results, and yet these genes were expressed at a lower level in spinal cords by day 21 p.i. (Fig. 5D to F). Expression of *Tmem173* was elevated in both monocytes/macrophages and DCs within brains at days 3 and 7 p.i., and an opposite trend was observed within spinal cords at day 21 p.i. (Fig. 5D to F). In contrast, *Tmem173* was constitutively expressed in microglia and expression was modified only slightly at days 3, 7, and 21 p.i. (Fig. 5E). Unlike microglia, both monocytes/macrophages and DCs expressed additional antiviral response factors *Irf1* (interferon regulatory factor 1) and *RipK1* at levels that were increased in brains at days 3 and 7 p.i. but were comparatively lower within spinal cords at day 21 p.i. (44, 45) (Fig. 5D to F). Finally, monocytes/macrophages also expressed *Irg1* (immune-responsive gene 1) at levels that were increased in brains at days 3 and 7 p.i. and lower in spinal cords at day 21 p.i. (Fig. 5D).

### IFN-γ pathways are enriched and associated with T cell-mediated control of JHMV replication by day 7 p.i.

**FIG 6:**
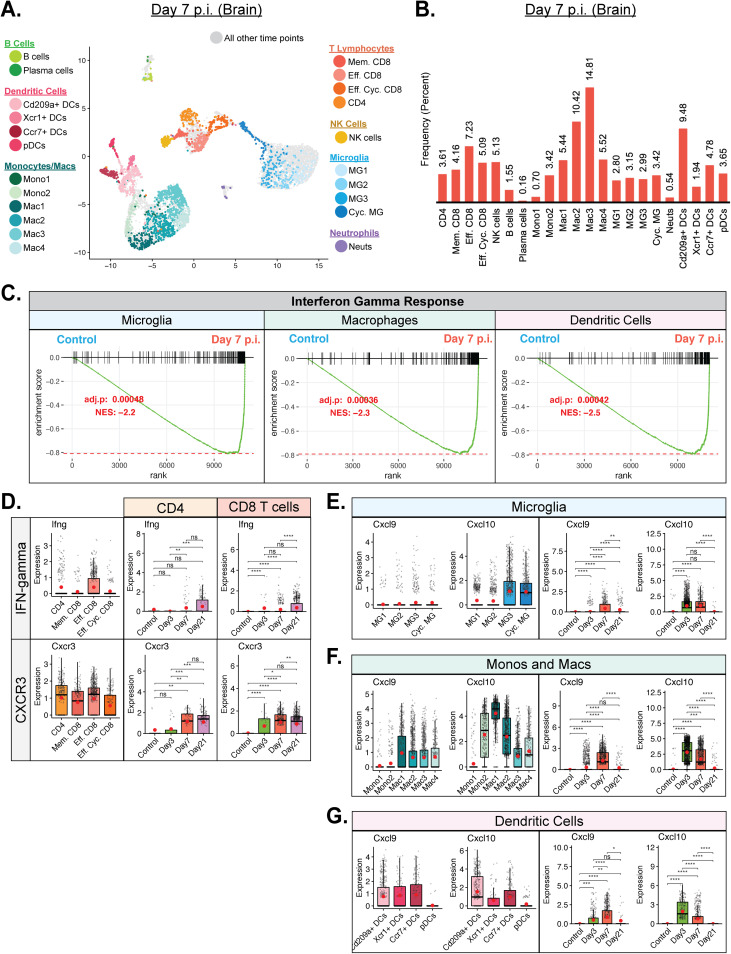
T cell infiltration into CNS correlates with enriched IFN-γ response. (A) UMAP plot showing cells within clusters exclusively from brains at day 7 p.i. with JHMV. (B) Bar graph showing frequency of cells from a cluster within the brains of mice 7 days postinfection (p.i.) with JHMV. By day 7 p.i., there was an increase in T cell populations as well as in macrophage and dendritic cell (DC) subsets. (C) Gene set enrichment analysis (GSEA) for IFN-γ responses in microglia, macrophages, and dendritic cells at day 7 p.i. from brains of JHMV-infected mice. The area under the curve represents enrichment of response genes. Normalized enrichment scores and *P* values are shown. (D) Expression of *Ifng* and *Cxcr3* transcripts by infiltrating T cell populations. (E to G) Expression of *Cxcl9* and *Cxcl10* transcripts in (E) microglia (MG), (F) subsets of monocytes (Mono) and macrophages (Mac), and (G) DC subsets. Temporal analysis results represent aggregate data from corresponding cell subsets. For the data presented in panels D to G, normalized expression values were used, and random noise was added. Box plots show interquartile range, median value (bold horizontal bar), and average expression value per sample (red dots). Wilcoxon’s test was used for statistical analysis. ns, not significant; *, *P* ≤ 0.05; **, *P* ≤ 0.01; ***, *P* ≤ 0.001; ****, *P* ≤ 0.0001.

By day 7 p.i., JHMV titers start declining within the brain and this coincided with entry of virus-specific T cells into the CNS that control viral replication. Consistent with this, infiltration of effector CD8^+^ T cells and effector cycling CD8^+^ T cells was increased at a greater proportion than was seen with CD4^+^ T cells and memory CD8^+^ T cells (Fig. 6A and B). We also detected an increase in Mac1, Mac2, Mac3, and Mac4 subpopulations as well as a selective increase in Cd209a^+^ DCs (Fig. 6A and B). GSEA revealed an increase in IFN-γ response genes in microglia, macrophages, and dendritic cells (Fig. 6C). Infiltrating CD4^+^ and CD8^+^ T cells expressed IFN-γ at all time points, with day 21 p.i. showing the highest level of expression (Fig. 6D). Overall, effector CD8^+^ T cells exhibited the highest levels of IFN-γ compared to other T cell subset populations (Fig. 6D). We and others previously demonstrated an important role for CXCL9 and CXCL10 in contributing to host defense in response to JHMV infection of the CNS by attracting virus-specific T cells and plasma cells into the CNS (15–17, 24, 46, 47). CXCR3 is the key signaling receptor for CXCL9 and CXCL10 and promotes migration of lymphocytes to the CNS in response to JHMV infection (48). *Cxcr3* transcripts were detected in both CD4^+^ and CD8^+^ T cell populations at levels that gradually increased from day 3 p.i. through day 21 p.i., reflecting the accumulation of T cells within the CNS of infected mice (Fig. 6D). Our earlier studies indicated that activated astrocytes were the primary cellular source for both CXCL9 and CXCL10 in response to JHMV infection (5, 15). Through scRNAseq, we were able to expand on these findings and show that subsets of microglia (Fig. 6E) as well as monocytes/macrophages (Fig. 6F) and DCs (Fig. 6G) were capable of expression of both *Cxcl9* and *Cxcl10* transcripts although expression was largely confined to days 3 and 7 p.i. within the brain and was not seen at day 21 p.i. within spinal cords.

### Differential expression of MHC transcripts within the CNS in response to JHMV infection.

**FIG 7:**
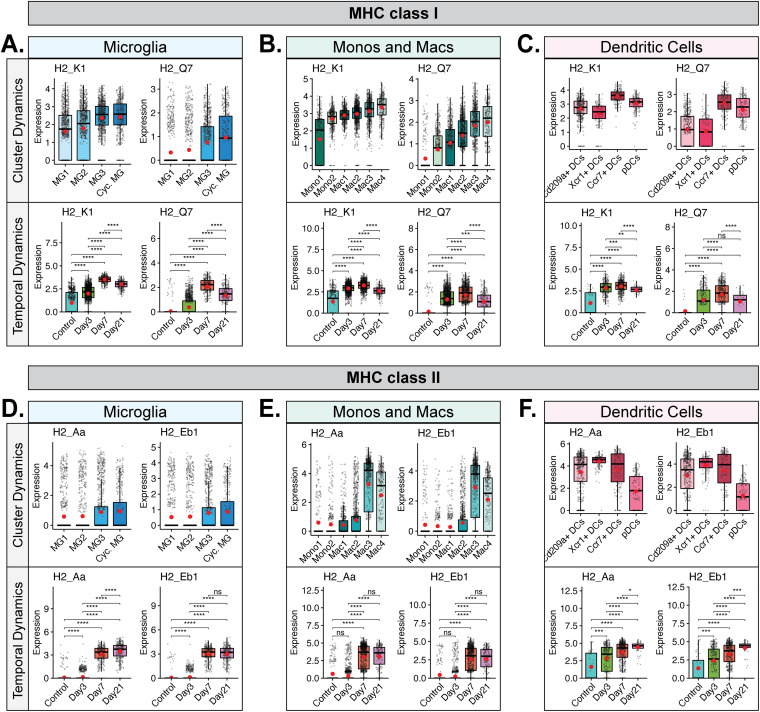
MHC and T cell inhibitor expression in microglia, monocytes/macrophages, and dendritic cells. (A to C) Box plots showing expression of MHC class I-associated genes *H2-K1* and *H2-Q7* in subpopulations of (A) microglia, (B) monocytes and macrophages, and (C) dendritic cells. (D to F) Expression of MHC class II-associated genes *H2-Aa* and *H2-Eb1* in subpopulations of (D) microglia, (E) monocytes and macrophages, and (F) dendritic cell subpopulations. In panels A to F, the top row demonstrates cluster dynamics and displays the aggregate data from all time points with regard to expression in subpopulations of cells, while the bottom row demonstrates temporal dynamics and shows overall expression of transcripts from the combined corresponding cell subtypes at defined times postinfection. Normalized expression values were used, and random noise was added. Box plots show interquartile range, median value (bold horizontal bar), and average expression value per sample (red dots). The Wilcoxon test was used for statistical analysis. ns, not significant; *, *P* ≤ 0.05; **, *P* ≤ 0.01; ***, *P* ≤ 0.001; ****, *P* ≤ 0.0001.

**FIG 8:**
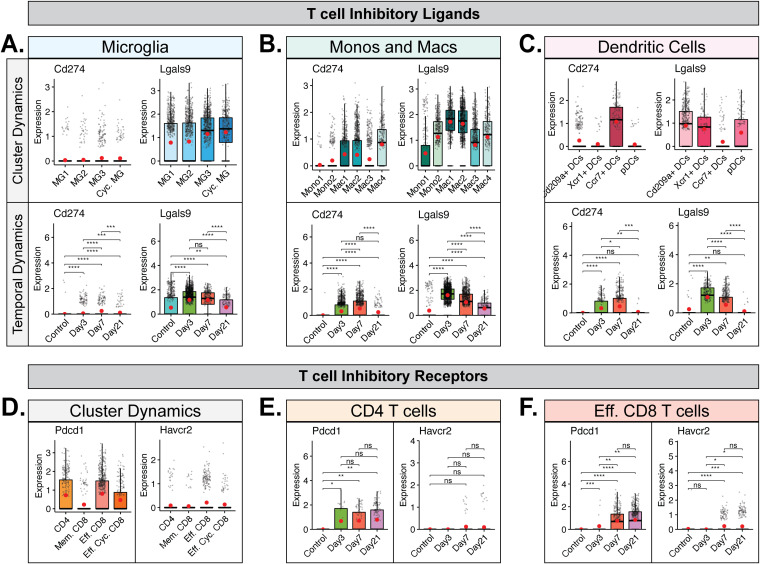
Expression of checkpoint inhibitor receptors and ligands in T cell populations and antigen-presenting cells. Box plots show expression of transcripts *Cd274* and *Lgals9* encoding T cell inhibitory ligands PD-L1 and Galectin-9 in subpopulations of (A) microglia, (B) monocytes and macrophages, and (C) dendritic cells. In panels A to C, the top row demonstrates cluster dynamics and displays the aggregate data from all time points with regard to expression in subpopulations of cells, while the bottom row demonstrates temporal dynamics and shows overall expression of transcripts from the combined corresponding cell subtypes at defined times postinfection. Panels D to F show expression of transcripts corresponding to T cell inhibitory receptors PD-1 (*Pdcd1*) and TIM-3 (*Havcr2*) in subpopulations of T cells. (D) Results of analysis of cluster dynamics of PD-1 and TIM-3 expression show the aggregate data from all time points with regard to expression in subpopulations of T cells. Results of temporal analysis of PD-1 and TIM-3 transcript expression are shown for (E) CD4^+^ T cells and (F) effector CD8^+^ T cells (data include Eff. CD8 and Eff. Cyc. CD8 clusters). Normalized expression values were used, and random noise was added. Box plots show interquartile range, median value (bold horizontal bar), and average expression value per sample (red dots). The Wilcoxon test was used for statistical analysis. ns, not significant; *, *P* ≤ 0.05; **, *P* ≤ 0.01; ***, *P* ≤ 0.001; ****, *P* ≤ 0.0001.

We next explored the transcript levels of MHC class I and II molecules in antigen-presenting cells (APCs) to evaluate population and temporal dynamics of antigen presentation to CD8^+^ and CD4^+^ T cells following viral infection with JHMV. We found that subsets of microglia, monocytes/macrophages, and dendritic cells expressed MHC class I-associated transcripts *H2-K1* and *H2-Q7* (Fig. 7A to C, cluster dynamics data). *H2-K1* transcripts were constitutively expressed in microglia, monocytes/macrophages, and dendritic cells in uninfected mice and subsequently increased following JHMV infection, showing highest expression at day 7 p.i. (Fig. 7A to C, temporal dynamics data). In contrast, transcripts for *H2-Q7* were expressed at almost undetectable levels in uninfected mice and yet increased after JHMV infection, also peaking at day 7 p.i. (Fig. 7A to C, temporal dynamics data). We observed an increase in transcripts encoding MHC class II-associated proteins, including *H2-Aa* and *H2-Eb1*, in subpopulations of microglia (Fig. 7D, cluster dynamics data), monocytes/macrophages (Fig. 7E, cluster dynamics data), and DCs (Fig. 7F, cluster dynamics data). Temporal analysis of these transcripts in microglia showed very low expression in uninfected mice that gradually increased by day 3 p.i. and that was dramatically elevated by days 7 and 21 p.i. (Fig. 7D, temporal dynamics data). In monocytes and macrophages, expression of MHC class II-associated transcripts was low in uninfected mice and remained so until it increased considerably at days 7 and 21 p.i. (Fig. 7E, temporal dynamics data). In DCs, there was constitutive expression of MHC class II-associated transcripts in uninfected mice that subsequently increased by days 3 and 7 p.i. and was highest at day 21 p.i. (Fig. 7F, temporal dynamics data). To further investigate the functional dynamics of APC populations following JHMV infection, we investigated the expression of transcripts encoding common T cell checkpoint inhibitory ligands, i.e., Programmed death-ligand 1 (PD-L1, *Cd274*) and Galectin-9 (*Lgals9*), to disclose any dampening of T cell function. Expression of PD-L1 transcripts was elevated in specific monocyte/macrophage and DC subsets but appeared negligible in microglia populations (Fig. 8A to C, cluster dynamics data). PD-L1 expression in these populations was also highest at days 3 and 7 p.i. (Fig. 8A to C, temporal dynamics data). Galectin-9 (Lgals9) expression appeared to range across a larger array of APC subsets (Fig. 8A to C, cluster dynamics data). Temporal analysis in microglia and monocytes/macrophages showed expression in uninfected mice that increased following JHMV infection and appeared highest at days 3 and 7 p.i. (Fig. 8A and B, temporal dynamics data). In DCs, Galectin-9 expression was also low in uninfected mice and increased at days 3 and 7 p.i., though expression had dropped by day 21 p.i. (Fig. 8C, temporal dynamics data). Looking at expression of transcripts encoding PD-L1 and Galectin-9 receptors PD-1 (*Pdcd1*) and TIM-3 (*Havcr2*) in T cells, we found that *Pdcd1* was primarily expressed in CD4^+^ and effector CD8^+^ T cell subsets (Fig. 8D). *Havcr2* (TIM-3) appeared to have been expressed in effector CD8^+^ T cells but at much lower levels than *Pdcd1* (Fig. 8D). In CD4^+^ T cells, *Pdcd1* expression was elevated at all defined times postinfection (Fig. 8E). In CD8^+^ T cells, expression of both *Havcr2* and *Pdcd1* was highest at days 7 and 21 p.i. (Fig. 8F).

### JHMV persistence is characterized with expression of molecules associated with disease and repair.

**FIG 9:**
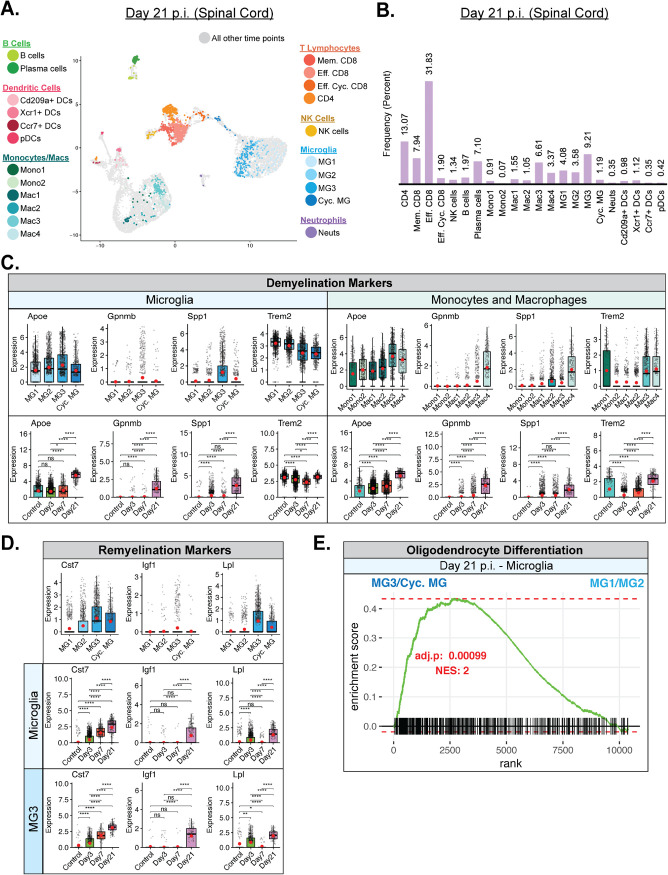
Microglia contribute to T cell and macrophage-mediated chronic demyelination and promote remyelination. (A) UMAP plot showing cells within clusters exclusively from spinal cords at day 21 p.i. with JHMV. (B) Bar graph showing frequency of cells from a cluster within the spinal cords of mice at day 21 postinfection (p.i.) with JHMV. By day 21 p.i., there was an increase in populations of T cells and plasma cells as well as specific macrophage and microglia subsets. (C) Expression levels of transcripts of the *Apoe*, *Gpnmb*, *Spp1*, and *Trem2* genes, which are associated with demyelination, were elevated in spinal cords at day 21 p.i. in specific subpopulations of microglia and macrophages. (D) Expression levels of remyelination markers *Cst7*, *Igf1*, and *Lpl* were also increased in specific subpopulations of microglia in spinal cords at day 21 p.i. with JHMV. For the data presented in panels C and D, temporal analysis results represent aggregate data from subsets. Normalized expression values were used, and random noise was added. Box plots show interquartile range, median value (bold horizontal bar), and average expression value per sample (red dots). Wilcoxon’s test was used for statistical analysis. (E) Gene set enrichment analysis (GSEA) of genes involved in promoting oligodendrocyte differentiation, comparing aggregated microglia subpopulations MG3 and cycling MG with MG1 and MG2 at day 21 p.i. from spinal cords of JHMV-infected mice. The area under the curve represents enrichment of involved genes. Normalized enrichment scores and *P* values are shown. ns, not significant; *, *P* ≤ 0.05; **, *P* ≤ 0.01; ****, *P* ≤ 0.0001.

By day 21 p.i., JHMV was found to have persisted within the spinal cords of surviving mice and this persistence was associated with ongoing neuroinflammation and demyelination in which inflammatory T cells and monocytes/macrophages contributed to white matter damage (8, 11, 13, 17, 49–51). scRNAseq performed on CD45^+^ cells isolated from spinal cords of infected mice at day 21 p.i. indicated that the prominent inflammatory cells consisted of T cell subsets, including CD4^+^ T cells, memory CD8^+^ T cells, and effector CD8^+^ T cells (Fig. 9A and B). In addition, expression levels of macrophage subset Mac3 and microglia subset MG3 were increased (Fig. 9A and B). Plasma cells were also identified, which is consistent with earlier studies demonstrating the importance of these cells in suppressing viral recrudescence (Fig. 9A and B) (24). We detected expression of transcripts of genes associated with demyelination, including apolipoprotein E (*Apoe*), transmembrane glycoprotein NMB (*Gpnmb*), osteopontin (*Spp1*), and triggering receptor expressed on myeloid cells 2 (*Trem2*) in various subsets of monocytes/macrophages and microglia (Fig. 9C) (52–54). While all microglia subsets expressed *Apoe* and *Trem2* transcripts, only MG3 expressed robust levels of *Spp1*; we did not detect *Gpnmb* transcripts in any microglia subpopulation (Fig. 9C). All of the monocyte/macrophage subpopulations expressed *Apoe*, while *Gpnmb* was enriched only in the Mac4 subsets, *Spp1* in the Mac2 and Mac4 subsets, and *Trem2* in the Mono1 subsets along with the Mac3 and Mac4 subsets (Fig. 9C). All DC subsets expressed *Apoe*, while only the Xcr1^+^ DC (also expressing the chemokine receptor for XCL1 and XCL2) expressed appreciable levels of *Trem2* transcripts (data not shown). Combining the data representing expression of specific transcripts from individual subsets, i.e., microglia and monocytes/macrophages, we observed an increase in transcript levels throughout the infection, with the highest levels being detected within spinal cords at day 21 p.i. (Fig. 9C). A notable exception to this was that expression of *Trem2* transcripts in microglia subsets gradually declined following JHMV infection compared to the transcript levels in control mice (Fig. 9C). Microglia subpopulations MG3 and Cyc. MG expressed transcripts encoding markers associated with remyelination, including Cystatin F (*Cst7*) (55, 56), insulin-like growth factor 1 (*Igf1*) (57, 58), and lipoprotein lipase (*Lpl*) (Fig. 9D) (59). Expression of *Cst7*, *Igf1*, and *Lpl* was highest in the MG3 population of microglia, followed by Cyc. MG (Fig. 9D). The highest levels of expression of *Cst7*, *Igf1*, and *Lpl* occurred within the spinal cord at day 21 p.i., and these findings support the notion that microglia may either directly or indirectly influence remyelination within the spinal cord by contributing to controlling expression of genes encoding proteins that regulate oligodendrocyte precursor cell (OPC) maturation (Fig. 9D) (60, 61). In further support, GSEA demonstrated enrichment of genes associated with oligodendrocyte differentiation by MG3 and Cyc. MG subpopulations compared to MG1 and MG2 subpopulations (Fig. 9E).

## DISCUSSION

Following i.c. inoculation of susceptible mice with JHMV, there is a rapid innate immune response that helps control viral replication until virus-specific T cells are able to expand and infiltrate the CNS. Virus-specific T cells control viral replication through IFN-γ secretion and cytolytic activity; however, virus is able to persist within the CNS primarily in white matter tracts, resulting in chronic neuroinflammation and demyelination, mediated primarily by T cells and macrophages. Significant strides have been made in understanding the mechanisms associated with both host defense and disease following JHMV infection of the CNS using bulk immune cells isolated from infected tissues. Given the complexity of the immune response to CNS viral infection, we employed scRNAseq as a powerful and high-resolution method to assess the molecular and cellular responses that aid in host defense, disease progression, and CNS repair. To accomplish this, we wanted to capture key events associated with the evolution of the immune response within the CNS during the innate, adaptive, and chronic stages of disease following JHMV infection. For analysis of innate and adaptive responses, we examined brains at days 3 and 7 p.i., respectively, as virus was beginning to replicate and expand within the brain at those times, with relative sparing of the spinal cord. By day 21 p.i., virus had spread into the spinal cord and animals had established immune-mediated demyelination and we chose that time to represent chronic disease. We used only uninfected brains to provide a baseline for subsequent comparison of immune responses within JHMV-infected brains and spinal cords via scRNAseq. While the brain and spinal cord are both within the CNS, we recognize these are not identical tissues and that using our approach did not capture events occurring within the spinal cord either prior to infection or at days 3 and 7 p.i. Nonetheless, we believe that our findings reveal the heterogeneity and complexity of immune response within the CNS following JHMV infection as the virus replicates and spreads within brains and spinal cords and uncover new pathways that may be contributing to demyelination as well as remyelination.

In the uninfected control brain, microglia subpopulation MG2 (27.61%) was the dominant CD45^+^ cell type present, followed closely by MG1 (16.41%) and the monocyte cluster Mono1 (16.41%) (Fig. 4B). All three of these clusters presented as a homeostatic counterpart to their overall defining cell types (32, 33). Microglia are considered to represent the immune cell of the CNS and are expected to be the primary CD45^+^ cells in the uninfected brain. The additional cell types present in the control brain included neutrophils (9.55%), B cells (5.95%), and Eff. Cyc. CD8 T cells (5.56%) (Fig. 4B). Our opinion is that these cells are present as a result of routine immune surveillance (40–43). We were surprised by the drop in the levels of neutrophils present within the brains from uninfected mice compared to infected mice as we had previously reported increased levels of neutrophils within the brains at day 3 p.i. with JHMV and that the levels subsequently declined as determined by flow cytometry (14). However, immature myeloid cells generally have gene expression signatures that overlap those of neutrophils and the two do not always neatly separate, especially considering the lack of differentiation occurring in the uninfected CNS, and we believe that this may have contributed to the surprisingly higher frequency of neutrophils found in the control group. By day 3 p.i., the most prominent cell types present within the brains included monocyte/macrophage and microglia subtypes, with the Mac2 subpopulation being the dominant cell type at that time. Compared to the other macrophage and monocyte subtypes, Mac2 was unique in that it showed increased expression of type I interferon response genes, including *Irf7*, *Srgn*, *Isg20*, and *Ifitm1* (Fig. 3C). This is consistent with GSEA showing an increase in IFN-α responses at day 3 p.i. in macrophages, microglia, and dendritic cells (Fig. 5C). Also note that, in conjunction with Mac2, the presence of unique subpopulations such as Mono2 (8.1%), Mac1 (9.0%), and Mac3 (5.9%) subpopulations at this time point suggests potential roles in host defense (Fig. 5B). Mac1 uniquely expressed transcripts for the chemokines *Cxcl10* and *Ccl4*, which attract NK cells, lymphocytes, and macrophages, respectively, as well as for *Rsad2*, which encodes the antiviral host protein Viperin (Fig. 5C). Mac3 represented the dominant antigen-presenting macrophage population, expressing MHC class II transcripts (*H2-Eb1*, *H2-Aa*, and *H2-Ab1*) and *Cd74* (Fig. 3C). In addition to the macrophage subpopulations present at that time point, microglia clusters MG1 (13.3%) and MG3 (14.7%) made up a large percentage at the day 3 time point, followed by MG2 (8.5%) and Cycling (Cyc.) MG (6.7%) (Fig. 5B). Of the top two microglia subpopulations, MG3 expressed proinflammatory chemoattractants encoded by *Ccl3*, *Ccl4*, and *Cxcl10*, similarly to the results seen with Mac1, as well as osteopontin (*Spp1*) and cholesterol 25-hydroxylase (*Ch25h*), while MG1 retained a homeostatic phenotype, with higher expression of *P2ry12*, *Slc2a5*, and *Gpr34* (Fig. 3D). In summation, the immune landscape of the CNS during the innate response to viral infection of the CNS with JHMV consists largely of microglia and macrophages that assume different roles involved in the type I IFN response and secretion of immune cell chemoattractants, while also reserving some cells for homeostatic maintenance.

At day 7 p.i., we found that the most prominent clusters were Mac3 (14.8%), Mac2 (10.4%), and Cd209a^+^ DCs (9.5%) (Fig. 6B). By that time, virus-specific T cells had expanded within the cervical lymph nodes and commenced trafficking into the CNS. Through scRNAseq analysis, we determined that the T cells infiltrating the CNS at day 7 p.i. were quite diverse and include cytolytic effector (Eff.) CD8^+^ T cells, Eff. Cyc. CD8^+^ T cells, and CD4^+^ T cells. A limitation of the current study was that astrocytes, which provide additional sources of chemokines, were not examined. We have previously reported that astrocytes are prominent cellular sources for CXCL9 and CXCL10 (5, 15–17). In the present study, we were able to determine that subpopulations of MG, monocytes, macrophages, and DCs expressed *Cxcl10* transcripts at days 3 and 7 p.i. This is in contrast to previously reported results for astrocyte-derived CXCL10, which was expressed at days 3 and 7 p.i. in the brain but also at day 21 p.i. in the spinal cord (5, 17). Expression of *Cxcl9* transcripts was primarily restricted to macrophage and DC subpopulations, with very little expression detected in either microglia or monocytes. Our earlier studies emphasized an important role for both CXCL9 and CXCL10 in attracting activated T cells and NK cells bearing the signaling receptor CXCR3 into the CNS of JHMV-infected mice during acute disease (15–17, 47, 48). Consistent with these earlier studies is the demonstration in this study that CNS-infiltrating subpopulations of T cells expressed *Cxcr3* transcripts. Infiltrating virus-specific T cells expressed IFN-γ upon recognition of cognate antigens expressed by activated antigen-presenting cells (APCs), and this aided in controlling JHMV replication as well as in enhancing expression of proinflammatory cytokines/chemokines that subsequently resulted in amplified neuroinflammation. Findings reported here demonstrate via GSEA an increase in expression of IFN-γ-responsive genes in microglia, macrophages, and dendritic cells, which correlated with increased expression of MHC class I and II transcripts within subpopulations of these cells (Fig. 6C; see also Fig. 7). For MHC class I-associated transcripts, there was an increase in expression within the brain at day 3 p.i. compared to uninfected mice in all examined cell populations and the expression levels gradually increased by day 7 p.i. and were sustained to day 21 p.i. in the spinal cord (Fig. 7A to C). For microglia and monocytes/macrophages, expression of MHC class II-associated transcripts was relatively low in the brains of both uninfected mice and infected mice at day 3 p.i. and yet dramatically increased at day 7 p.i., coinciding with the infiltration of virus-specific T cells expressing IFN-γ (Fig. 7D and E). Expression of MHC class II transcripts in these APC populations remained elevated in the spinal cords to day 21 p.i. (Fig. 7D and E). Within activated DC subpopulations, we detected higher basal levels of MHC class II-associated transcripts from uninfected mice that gradually increased following JHMV infection to day 21 p.i., supporting results of earlier studies indicating that JHMV exhibits preferential infection of mature DCs (Fig. 7F) (62). The unique temporal expression profiles of MHC molecules between APC populations suggest an intricately orchestrated system of antiviral defense in which different cell populations employ the same functions consecutively and not simultaneously, with dendritic cells and monocyte/macrophage populations presenting antigen more acutely and microglia subtypes later in disease. Similarly, surveying expression of transcripts encoding two common T cell checkpoint inhibitory ligands, Programmed death-ligand-1 (PD-L1, *Cd274*) and Galectin-9 (*Lgals9*), we found that DCs had a more limited role later in disease, with reduced expression at day 21 p.i. (Fig. 8C). Microglia and monocytes/macrophages expressed *Lgals9* (Galectin-9) in uninfected mice, but expression levels were higher following infection with JHMV and persisted to day 21 p.i. in spinal cords (Fig. 8A and B). *Cd274* (PD-L1) was expressed primarily in Mac1, Mac2, and Mac4 macrophage populations and the Ccr7^+^ DC population, with almost no expression seen in microglia (Fig. 8A to C). Programmed cell death-1 (PD-1) and T-cell immunoglobulin and mucin domain-containing-3 (TIM-3) are inhibitory receptors that are present on activated T cells, where binding to the corresponding ligands, PD-L1 and Galectin-9, results in transmission of an inhibitory signal with respect to T cell effector function (63, 64). Furthermore, the presence of these receptors and ligands suggested T cell exhaustion (64, 65). To determine if T cells would show similar temporal expression of the corresponding inhibitory receptors, we evaluated expression of transcripts encoding PD-1 (*Pdcd1*) and TIM-3 (*Havcr2*) in T cells. We found that *Pdcd1* (PD-1) was primarily expressed in CD4^+^ T cells and effector CD8^+^ T cell subsets, while *Havcr2* (TIM-3) was expressed mostly by effector CD8^+^ T cells and at much lower levels (Fig. 8D). *Pdcd1* (PD-1) expression was elevated at all defined times postinfection in CD4^+^ T cells, but expression was highest at days 7 and 21 p.i. in effector CD8^+^ T cells (Fig. 8E and F). Similarly, the levels of expression of transcripts encoding TIM-3 (*Havcr2*) were also elevated at days 7 and 21 p.i. in effector CD8^+^ T cells (Fig. 8F). These findings argue that effector CD4^+^ and CD8^+^ T cells exhibit an exhausted phenotype at later stages of disease that may impact control of viral replication (23).

Following the acute phase of infection, virus begins to spread into the mouse spinal cord and this is associated with immune-mediated demyelination. By day 21 p.i., peak inflammation has subsided and infected mice are left with a chronic, demyelinating spinal cord lesion. Inflammatory T cells and macrophages are known to contribute to this demyelination (8, 11, 17, 66–70), although the mechanisms by which this occurs remain weakly characterized. To contribute further insight into the physiological players that enhance or control white matter damage during the chronic stage of infection, we conducted scRNAseq analysis of CD45^+^ cells present within the spinal cord at day 21 p.i. We found enriched populations of CD4^+^ T cells, effector CD8^+^ T cells, memory CD8^+^ T cells, and plasma cells (Fig. 9A and B). Among the macrophage and microglia populations found in the spinal cord at day 21 p.i., Mac3 and MG3 appeared to be dominant (Fig. 9A and B). Evaluating expression of demyelination-associated transcripts encoding APOE protein (*Apoe*), NMB transmembrane glycoprotein (*Gpnmb*), osteopontin (*Spp1*), and TREM2 (*Trem2*) in microglia and macrophage subpopulations (52–54), we found that MG3, Mac3, and Mac4 showed the highest expression compared to other subpopulations (Fig. 9C). Furthermore, the levels of expression of these transcripts in microglia and macrophages were elevated at day 21 p.i. (Fig. 9C). These findings are consistent with our previous work showing elevated levels of transcripts of *Apoe*, *Spp1*, and *Trem2* in spinal cords at an earlier stage of demyelination (26) and further support the notion that these cells tailor the spinal cord microenvironment to regulate ongoing disease. Defining potential roles for these factors in augmenting demyelination is the focus of ongoing studies. As microglia are known to assume a myriad of functional roles, we sought to determine if any subpopulations present in chronically demyelinated lesions have the potential to ameliorate white matter pathology by evaluating expression of transcripts of *Cst7* (Cystatin F) (55, 56), *Igf1* (Insulin-like growth factor-1, IGF-1) (57, 58), and *Lpl* (lipoprotein lipase) (59), which are associated with promoting remyelination, and found that the levels of the transcripts were highest in the MG3 subpopulation of microglia, with expression also elevated in Cyc. MG (Fig. 9D). The levels of expression of remyelination-associated transcripts were also found to be the highest in spinal cords at day 21 p.i. (Fig. 9D). However, expression of *Cst7*, *Igf1*, and *Lpl* appeared to be more relevant to the later stages of spinal cord demyelination seen at day 21 p.i. than our previous findings obtained by examining an earlier stage of demyelination in spinal cords (26). Furthermore, using GSEA to compare overall levels of transcripts associated with promoting oligodendrocyte differentiation between MG3/Cyc. MG and MG1/MG2, we found that MG3/Cyc. MG showed a drastic increase in comparison at day 21 p.i. (Fig. 9E). Collectively, these findings reinforce the concept that different populations of microglia and macrophages exist within the inflamed CNS during neuroinflammatory disease that can both affect disease progression and repair damage and may offer targets for interventional therapy (26, 53, 71–74).

## MATERIALS AND METHODS

### Mice and viral infection.

C57BL/6 male mice (5 weeks of age) were purchased from The Jackson Laboratory. Mice were infected intracranially (i.c.) with 1,500 PFU of JHMV strain J2.2v-1 in 30 μl of sterile Hanks balanced sterile solution (HBSS), and animals were euthanized using isoflurane and were perfused with phosphate-buffered saline (PBS) at days 0 (uninfected control), 3, 7, and 21 postinfection (p.i.). Clinical disease in JHMV-infected mice was evaluated using a previously described scale (68). One half of each brain was used for determining viral titers, and the other half was used for scRNAseq. Spinal cords obtained at day 21 p.i. were used for determining either viral titer or scRNAseq. Isolated brains and spinal cords were homogenized and plaque assays were performed on the DBT astrocytoma cell line as described previously (75). All animal studies were reviewed and approved by the University of Utah Animal Care and Use Committee.

### Cell isolation and single-cell RNA sequencing (scRNAseq).

To isolate immune cells from brains (uninfected controls; day 3 and day 7 p.i.) and spinal cords (day 21 p.i.), mice were anesthetized using isoflurane and were perfused with PBS. Brains and/or spinal cords were removed, and single-cell suspensions were generated from tissue samples by grinding using frosted microscope slides in 5 ml of RPMI medium as described previously (68). Immune cells were then enriched via the use of a 2-step Percoll cushion (90% and 63%), and cells were collected at the interface of the two Percoll layers (76, 77). Cells were stained with DAPI (4′,6-diamidino-2-phenylindole) and allophycocyanin-conjugated anti-CD45 for 20 min on ice in 1× PBS–0.5% bovine serum albumin (BSA). Live CD45^+^ cells were enriched through the use of a BD Aria fluorescence-activated cell sorter (FACS) (University of Utah Health Science Center) and were washed once with 0.04% BSA. Samples were then processed for single-cell RNA sequencing via the use of the 10X Genomics platform at the Huntsman Cancer Institute High Throughput Genomics Shared Resource Core Facility (https://uofuhealth.utah.edu/huntsman/shared-resources/gba/). An Agilent HiSeq next-generation sequencer was used to perform RNA sequencing (125 cycles), and sequencing reads were processed using the 10X Genomics CellRanger pipeline. Using the Seurat R package, unwanted cells were filtered out based upon mitochondrial gene representation and variances in unique gene counts. Downstream analysis was performed using the Seurat package following principal-component analysis (PCA) and uniform manifold approximation and projection (UMAP) dimensional reduction (27, 28). Gene expression signatures of cells from brains (uninfected controls, day 3 and day 7 p.i.) and spinal cords (day 21 p.i.) of JHMV-infected mice were scrutinized, and cells from the aggregated data set were clustered into corresponding immune cell populations by the use of a shared nearest neighbor (SNN) modularity optimization-based clustering algorithm via the FindClusters function in the Seurat analysis package in R. The resulting clusters were defined using an immune cell scoring algorithm (https://aekiz.shinyapps.io/CIPR/) (29, 30) that compares the gene signatures of each cluster in the experimental data set with the microarray data available in the Immunological Genome (ImmGen) Project Database (29, 30). Expression levels and distributions of population-specific immune cell markers were then analyzed to further identify subpopulations. Once the clusters were established and identified, plots were generated using Seurat and ggpubr R packages and GSEA plots were generated as previously mentioned using the fgsea R package (30).

### Histology.

Mice were euthanized at defined time points according to IACUC-approved guidelines, and the length of spinal cord extending from thoracic vertebrate 6 to vertebrate 10 was cryoprotected in 30% sucrose, cut into 1-mm transverse blocks, processed so as to preserve the craniocaudal orientation, and subsequently embedded in optimum cutting temperature (O.C.T.) compound (VWR, Radnor, PA, USA). Coronal sections (8 μm thick) were cut, and the sections were stained with hematoxylin/eosin (H&E) in combination with Luxol fast blue (LFB) (18, 78, 79).

### Statistical analysis.

GraphPad Prism was used to perform statistical analyses. Data for each experiment are presented as means + standard errors of means (SEM). The Wilcoxon test was used for analyzing gene expression in scRNAseq clusters, and the resulting *P* values were corrected for multiple comparisons by the Holm-Sidak method. A *P* value of ≤0.05 was considered statistically significant (*, *P* ≤ 0.05; **, *P* ≤ 0.01; ***, *P* ≤ 0.001; ****, *P* ≤ 0.0001).
